# Laparoscopic Repair of Bilateral Congenital Diaphragmatic Hernia Without Pulmonary Hypoplasia

**DOI:** 10.7759/cureus.10335

**Published:** 2020-09-09

**Authors:** Mehreen Mahmood, Teerin Meckmongkol, Tamarah Westmoreland

**Affiliations:** 1 Emergency Medicine, University of Central Florida College of Medicine, Orlando, USA; 2 Pediatric Surgery, Nemours Children's Hospital, Orlando, USA; 3 Surgery, University of Central Florida College of Medicine, Orlando, USA

**Keywords:** bilateral diaphragmatic hernia, laparoscopy, pulmonary hypoplasia, congenital diaphragmatic hernia, laparascopic repair

## Abstract

We report the case of a 14-month-old female who had a right-sided congenital diaphragmatic hernia (CDH) without pulmonary hypoplasia. The patient was preoperatively diagnosed with a Morgagni hernia due to the size and location of the hernia seen on imaging. However, the patient was found to have bilateral diaphragmatic defects intraoperatively, and her right diaphragm was almost completely absent. Our patient did not have pulmonary hypoplasia or any of the respiratory comorbidities that CDH patients typically present with, though she did have repeated respiratory infections and cough. This case demonstrates that CDH is not always diagnosed in an accurate or timely manner radiographically and that the surgeon should be prepared to potentially repair more of the diaphragm than expected. Additionally, there is a need to study the pathophysiology and genetics of CDHs further.

## Introduction

The diaphragm is a mesodermal structure that develops around weeks 4-10 of human development [1]. The diaphragm develops from an unpaired ventral portion (the septum transversum), from paired dorsal lateral portions (pleuroperitoneal folds), and from an irregular medial dorsal portion (dorsal mesentery) [1-3]. The pleuroperitoneal folds fuse with the septum transversum, and the esophageal mesentery and muscular ingrowth from the body wall invade the folds, forming the muscular part of the diaphragm. If any of these parts do not fuse correctly, a hernia may form [2,4].

Congenital diaphragmatic hernias (CDHs) are rare congenital anomalies of the diaphragm with an incidence of approximately 1:2,500 births [3-8]. CDHs are classified into two groups based on the anatomic position of the defect: posterolateral or anterior/central [3]. The posterolateral (Bochdalek) occurs in 70%-75% of cases. These hernias are most commonly found on the left (85%), but they may also be on the right (13%) or even bilaterally (2%) [1,3,4].

The pathogenesis of CDH is not universally agreed upon. Classically, it was believed that the initial defect was in the diaphragm and then once the abdominal organs herniated into the thoracic cavity, the lungs failed to develop properly. The pulmonary hypoplasia and abnormal pulmonary vascular development was thus deemed as the second defect [1,4]. However, the introduction of the nitrofen animal model for CDH by Keijzer et al. has led to a new ‘dual-hit’ hypothesis [5]. Essentially their research showed that pulmonary hypoplasia occurred before the diaphragm failed to close. This was shown because regardless of which side the diaphragmatic defect was on, many patients had bilateral pulmonary hypoplasia. So, the dual hit hypothesis states that the initial insult is bilaterally within the lungs themselves prior to the diaphragms development and the second insult is in the ipsilateral lung due to abdominal content herniation [1,5,9].

The majority of CDH cases are diagnosed prenatally through ultrasonography [8]. The diagnostic finding is the presence of abdominal organs in the thoracic cavity, especially for left-sided defects. Right-sided hernias are more likely to be missed because the liver tends to be the only organ to herniate into the thoracic cavity initially, and it has a similar echogenicity to the lungs on ultrasound [3,6,8]. Additionally, liver herniation tends to worsen prognosis in patients due to the implied larger defect as well as the implication of concomitant pulmonary hypoplasia and pulmonary hypertension [9].

Late presentation of CDHs is expected to have a better prognosis, usually because the defect is not large enough to lead to compression of the developing lung [10]. Therefore, the baby has sufficient pulmonary function to sustain life. These patients have clinical symptoms of either recurrent respiratory symptoms or gastrointestinal symptoms [11]. Typically, an older child presents with gastrointestinal symptoms of CDH due to incarceration of parts of the digestive system [11,12]. Patients with milder cases may not present with respiratory or gastrointestinal symptoms for several months or years into life [2,12].

We present the case of a 14-month-old female who underwent repair of her diaphragmatic hernias. She was initially diagnosed with a right-sided CDH, but, during surgery, she was discovered to have bilateral diaphragmatic hernias, and her right-sided defect was significantly larger than seen via imaging.

## Case presentation

A 14-month-old female presented to Nemours Children's Hospital secondary to a respiratory infection and a concern for a Morgagni hernia. She was born at 37 weeks' gestation via spontaneous vaginal delivery without any prenatal, postnatal, or perinatal complications reported. She did not have any episodes of cyanosis or increased work of breathing at birth. At about eight days of age, she was diagnosed with a large membranous ventricular septal defect (VSD) with inlet extension measuring approximately 7 mm diameter with bidirectional flow, a small fenestrated secundum atrial septal defect, and a small patent ductus arteriosus. 

When the patient was four months old, she presented to an ED after having cough, congestion, and wheezing for four to five days. A chest x-ray at that time noted a right anterior diaphragmatic hernia.

**Figure 1:**
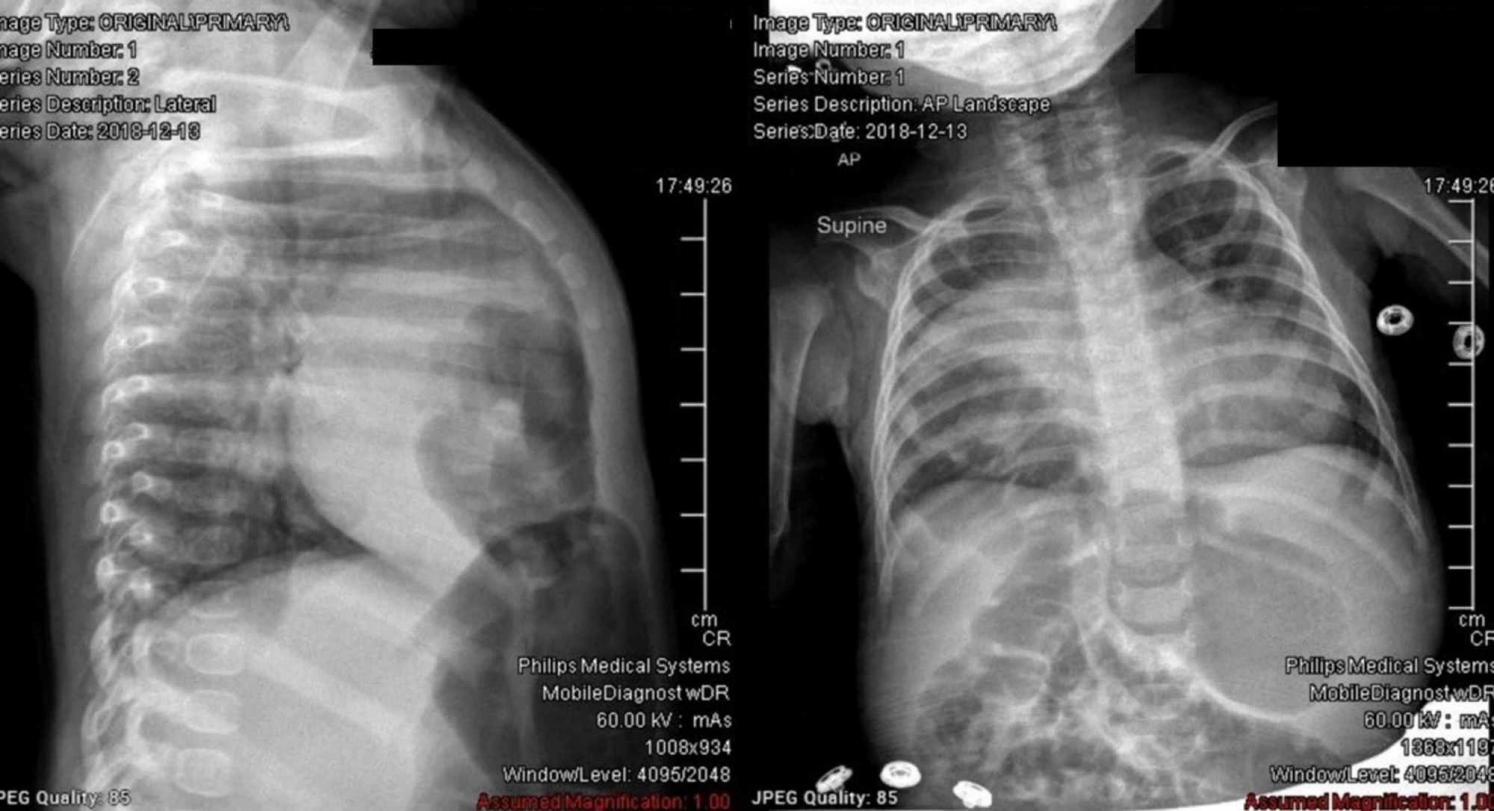
Lateral (on right) and anteriorposterior (on left) chest X-ray showing herniated colonic loops in the anterior right hemithorax consistent anterior right diaphragmatic hernia

She was then transferred to the Nemours Children’s Hospital where general surgery was consulted. The patient was diagnosed with a likely Morgagni’s type hernia based on her chest CT scan and her history of productive cough for five days.

The CT report stated that there was evidence of an anterior right diaphragmatic hernia, noting herniated colonic loops in the anterior right hemithorax. The right lung had some associated compressive atelectasis adjacent to the herniation. The hepatic borders appeared to remain below the right hemidiaphragm.

At her initial presentation of respiratory distress, she was found to have a viral illness; therefore, the surgical team elected to wait till the patient was closer to 12 months of age before repairing her CDH. Furthermore, she was below weight and needed improved nutrition prior to surgery. Moreover, she was evaluated at the time for potential repair of her VSD.

The patient was re-evaluated in the surgery clinic at seven months of age for a surgical date, and her weight was still below the third percentile. The surgeon decided that the patient could be scheduled for a surgery after she was cleared by the cardiology team for her VSD. Her cardiologist and cardiac surgeon both deemed her VSD stable and in no need for a repair. She was then scheduled for a repair when she would be 14 months of age.

At the time of her procedure, the patient weighed 7.226 kg. Her mother denied any recent illnesses, fever, nausea, vomiting, or diarrhea. On physical exam, she was alert and not in distress. Head, eyes, ears, nose, throat (HEENT) exam was unremarkable. She had unlabored respirations, no intercostal retractions, or accessory muscle use, and her lungs were clear to auscultation bilaterally without any rales or wheezes. Heart was regular rate and rhythm with a grade 2-3/6 holosystolic murmur heard at the midsternal border. Her abdomen was soft, non-tender, non-distended without organomegaly and with normal bowel sounds.

The patient underwent laparoscopic repair of her defect. The initial 5-mm port was placed in the infraumbilical position with two additional 5-mm ports placed in the left and right upper quadrants. The patient was noted to have a very large right-sided diaphragmatic hernia that contained both colon as well as the left and right lobes of the liver. While she did have a small area of normal diaphragmatic tissue, the rest of her right diaphragm was completely absent. The right diaphragmatic hernia was in fact greater than twice the size of a normal Morgagni hernia. A portion of the right diaphragmatic hernia sac was attempted to be removed; however, this was abandoned because it partially opened the right pleura. The right pleura was then widely open to prevent a tension pneumothorax. She was noted to have a developed right lung. Additionally, the patient was found to have a small, 4-cm left-sided diaphragmatic hernia.

Both defects were repaired via interrupted 3-0 Ethibond surtures (Ethicon, Somerville, NJ). The patient maintained excellent hemostasis throughout the procedure. An intraoperative chest x-ray was performed. This revealed a mild right-sided pneumothorax secondary to opening the right pleura. Because the patient was hemodynamically stable and doing quite well, a chest tube was not placed in the right chest.

**Figure 2:**
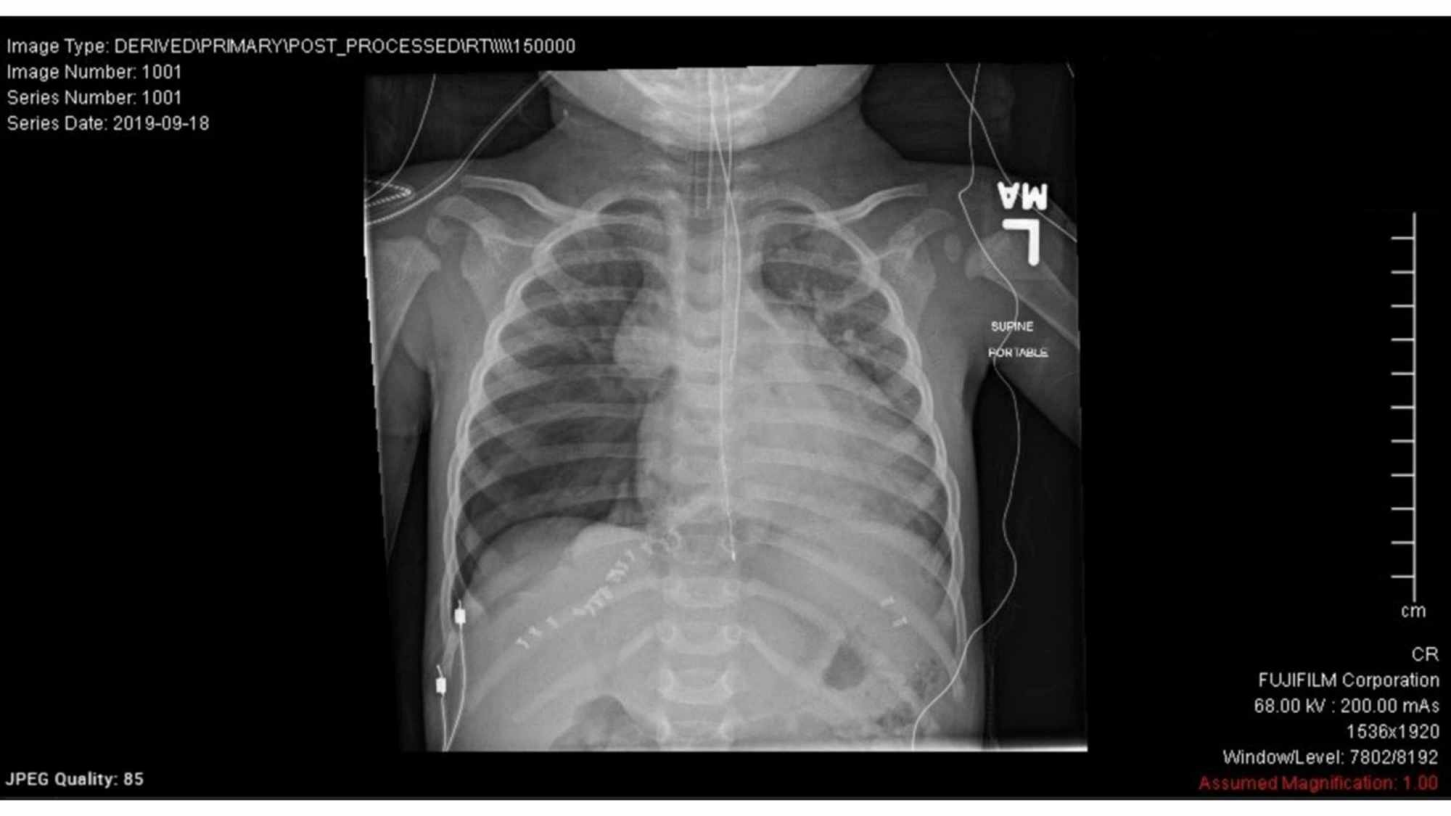
Intraoperative X-ray showing moderate right-sided pneumothorax status post hernia repair

The patient did very well overnight at the hospital. On physical exam on postoperative day 1, she was alert, not in distress and smiling. Her lungs were clear to auscultation bilaterally without rales or wheezes; no intercostal retractions or accessory muscle use was found. Her heart was regular rate and rhythm. She was discharged that day without any problems. 

## Discussion

Only 2% of CDH malformations present with bilateral defects [1,4]. We present this patient to provide another case of this rare anomaly with an unusual presentation.

Despite the patient’s mother being compliant with her prenatal ultrasounds, the diagnosis of CDH was not made until after birth. This shows how easy it is to miss a right-sided diaphragmatic hernia on a prenatal ultrasound [13]. As stated earlier, right-sided defects are more difficult to detect. While left-sided defects lead to herniation of abdominal contents into the thoracic cavity, which is easier to see on ultrasound, right-sided defects only have liver herniation which is of similar echogenicity to the lungs and thus, harder to spot on ultrasound [4,13]. Color Doppler ultrasound can be helpful in determining liver position by visualizing the ductus venosus and the intrahepatic vessels [4]. MRI is the easiest way of assessing liver herniation [13]. Unlike ultrasound, maternal obesity or oligohydramnios does not affect findings, and MRI better demonstrates soft tissue contrast and is better at detecting comorbidities [4,13].

Our understanding of CDH is that the larger the defect, the more likely the patient will have pulmonary hypoplasia and respiratory distress [8]. This is because with a defect in the diaphragm, abdominal organs can herniate through it and into the thoracic cavity. This then leads to impairment of pulmonary development [4]. Additionally, the dual hit hypothesis postulates that all patients with CDH have bilateral pulmonary hypoplasia and abnormal pulmonary vascular development regardless of the size or positioning of the hernia [9,13].

There is increasing evidence that CDH pathogenesis has genetic causes. Approximately 40% of CDH cases have associated congenital abnormalities [1,6,14]. There are many genetic syndromes and chromosomal abnormalities also associated with diaphragmatic defects [10]. Outside of pulmonary issues, the most common group of congenital anomalies is of cardiovascular origin. Specifically, 11%-15% of CDH patients also present with cardiovascular anomalies [6,13,14].

## Conclusions

Our patient had cardiovascular malformations that were being evaluated. This patient is unique in that she had a very large right-sided CDH with no pulmonary hypoplasia likely secondary to the liver protecting her right thoracic cavity from her intra-abdominal organs, thereby allowing her pulmonary tissue to develop. While she did have respiratory distress at four months of age and had a history of respiratory illnesses throughout her life, her lungs appeared to be normal on all imaging modalities. Additionally, during surgery her right lung was examined and appeared well developed. Our patient was discharged from the hospital the day after her surgery, so she was in remarkable health despite her defects.

Our patient’s case demonstrates that further studies in the pathogenesis and development of CDHs are needed. While her associated cardiovascular anomaly shows that there are likely genetic components to CDH, identifying these genetic and/or environmental influences has yet to be elucidated.
